# Dietary Inflammatory Potential in relation to General and Abdominal Obesity

**DOI:** 10.1155/2022/5685249

**Published:** 2022-01-31

**Authors:** Saeedeh Nouri-Majd, Asma Salari-Moghaddam, Ammar Hassanzadeh Keshteli, Ahmad Esmaillzadeh, Peyman Adibi

**Affiliations:** ^1^Department of Community Nutrition, School of Nutritional Sciences and Dietetics, Tehran University of Medical Sciences, Tehran, Iran; ^2^Department of Medicine, University of Alberta, Edmonton, Alberta, Canada; ^3^Integrative Functional Gastroenterology Research Center, Isfahan University of Medical Sciences, Isfahan, Iran; ^4^Department of Community Nutrition, School of Nutrition and Food Science, Isfahan University of Medical Sciences, Isfahan, Iran; ^5^Obesity and Eating Habits Research Center, Endocrinology and Metabolism Molecular-Cellular Sciences Institute, Tehran University of Medical Sciences, Tehran, Iran

## Abstract

*Background*/Aims: Limited data are available on the association of Dietary Inflammatory Potential (DIP) with general and abdominal obesity in developing countries. The aim of this study was to examine the association between DIP score with general and abdominal obesity among Iranian adults. *Methods*. This cross-sectional study was conducted among adults in Isfahan, Iran. Dietary intakes were assessed by using a validated, self-administrated, dish-based, semiquantitative food frequency questionnaire. DIP was calculated based on standard method. Data regarding height, weight, and waist circumference (WC) were collected using a self-reported questionnaire. Overweight or obesity was defined as body mass index (BMI) ≥25 kg/m^2^, and abdominal obesity was defined as WC ≥ 80 cm for women and ≥94 cm for men. *Results*. Mean age of study participants was 36.8 ± 8.08 years. The prevalence of general and abdominal obesity was 46.5% and 52.9%, respectively. We observed that higher DIP scores were significantly associated with a lower odds of general obesity (OR: 0.66; 95% CI: 0.58–0.74). Stratified by sex, this significant association was seen only for women (OR: 0.58; 95% CI: 0.46–0.72). In addition, no significant association was found between DIP scores and abdominal obesity. *Conclusions*. We found a significant inverse association between consumption of a proinflammatory diet and general obesity. In the gender-stratified analysis, this was seen in women, but not in men. There was no significant association between the DIP scores and abdominal obesity.

## 1. Introduction

Obesity is an inflammatory condition, in which circulating inflammatory biomarkers are elevated [1]. Dietary Inflammatory Potential (DIP) is a new tool to assess inflammatory potential of an individual's diet [2]. Earlier studies examined the association of DIP with various diseases including cardiovascular disease [3], many types of cancers [4], and metabolic syndrome [5]. In a cohort study on the Spanish population, proinflammatory diet, as measured by DIP, was significantly associated with developing overweight or obesity [6]. This was also reported in a cross-sectional study [7].

Obesity pattern in these countries seems to be different from those in other nations. In addition, abdominal obesity is more prevalent in these countries and among women than that in men [8]. On the other hand, diet also seems to be different in this part of the world. A cross-sectional study in Iran showed that the dietary inflammatory index was associated with enlarged waist circumstance [9]. This was in line with another study that demonstrated elevated levels of inflammatory cytokines in general and abdominal obesity [10]. Some investigators believe that inflammatory cytokines are responsible for insulin resistance in obesity [11] while some animal studies showed that proinflammatory cytokines can increase thermogenic gene expression and energy expenditure and may play a role in preventing obesity [12, 13]. This was also confirmed by another investigation in which administration of TNF-*α* inhibitors resulted in an increased body weight and BMI [14]. Given these controversies about the role of inflammation in obesity, it seems that additional studies are needed to shed light on this issue. The aim of the present study was to examine the association of a proinflammatory diet and general and abdominal obesity in a large sample of Iranian adults.

## 2. Methods and Materials

Participants: this cross-sectional study was conducted based on the comprehensive study “Epidemiology of Psychological, Alimentary Health and Nutrition (SEPAHAN)” which was implemented in 50 different healthcare centers throughout Isfahan province, affiliated to Isfahan University of Medical Sciences (IUMS) between April 2010 and May 2010. More information about the SEPAHAN project in our previous study was published [15]. In this project, information about demographic factors, anthropometric indices, and lifestyle factors including physical activity and diet was collected using a self-administered questionnaire that 8,691 people returned completed questionnaire. In this study, people who did not have data on each relevant variable and people who had a total daily energy intake of less than 800 or more than 4200 kcal per day were excluded. After excluding these people, 6,724 adults (3,127 (46.5%) individuals with general obesity) were considered in our analyses for general obesity and 5,219 adults (including 2,763 (52.9%) people with abdominal obesity) were included in the analysis of abdominal obesity. The different number of people in the analyses of general and abdominal obesity was due to lacking data on waist circumference for some individuals. Written consent forms were given to participants and all of them submitted the form. The study protocol was approved ethically by the Regional Bioethics Committee of Isfahan University of Medical Sciences.

Dietary intakes assessment: to gather dietary data, the Willett-format dish-based 106-item semiquantitative food frequency questionnaire (DS-FFQ) was used. This questionnaire is specifically designed and validated for Iranian adults. Further details on this questionnaire have already been published [16]. In summary, this questionnaire consisted of five categories of foods and dishes: (1) miscellaneous food items and beverages (including fast foods, sweets, desserts, beverages, and nuts, 36 items); (2) fruits and vegetables (22 items); (3) grains (potato, different types of bread, biscuits, and cakes, 10 items); (4) dairy products (cream, butter, and dairies, 9 items); and (5) mixed dishes (canned or cooked, 29 items). The “household measures” booklet was used to calculate the amounts of foods consumed [17]. In a study of 200 randomly selected subjects, the validity of the questionnaire was examined. The questionnaire was completed by all participants twice, at the beginning of the study and after 6 months. Three detailed dietary records were also completed by participants. Comparison of questionnaire data and average dietary records showed that this questionnaire works well in estimating long-term dietary intakes [16].

Assessment of DIP: DIP score calculations were performed using dietary data derived from the DS-FFQ. The development and validation of DIP have been investigated in previous studies [18–20]. Findings from the study by Shivappa et al. showed an association between 45 nutrients and specific foods with one or more of the inflammatory biomarkers (CRP, Interleukin-6 (IL-6), Tumor Necrosis Factor-*α* (TNF-*α*), or Interleukin-1*β* (IL-1*β*)) or anti-inflammatory biomarkers (Interleukin-10 (IL-10) and Interleukin-4 (IL-4)) [20]. Then, they determined the inflammatory potential for each food parameter according to the following, it declined inflammatory or reduced anti-inflammatory factors (+1), it improved inflammatory or increased anti-inflammatory factors (−1), and ineffective on inflammatory or anti-inflammatory biomarkers (0). They used data from 11 datasets from 11 countries around the world to calculate the global mean and standard deviation for each of the 45 food parameters. Due to missing some items (like polyphenols) in our nutrient database and the lack of consumption of some foods in Iranian dietary culture, in the current study, we calculated the DIP score based on 29 food parameters (rather than 45). In the present study, we used the following food parameters: proinflammatory parameters included energy, protein, fat, carbohydrate, saturated fat, cholesterol, trans fat, iron, and vitamin B12 and anti-inflammatory parameters included polyunsaturated fatty acids (PUFA), monounsaturated fatty acids (MUFA), fiber, thiamin, riboflavin, niacin, vitamin B6, folic acid, vitamin C, vitamin A, *β*-carotene, vitamin E, vitamin D, selenium, magnesium, zinc, pepper, onion, caffeine, and tea. First, we calculated energy-adjusted amounts of these nutrients using the residual method [21]. Then, the *z* score for a given food parameter was calculated by subtracting the global standard mean from the amount consumed by each participant and dividing it by the global standard deviation and based on that, the DIP score was calculated for each participant. Global means and standard deviations were obtained from the study of Shivappa et al. [20]. We converted this value to a centered percentile score in order to reduce skewness, as earlier studies did [20]. For each person, this score was multiplied by the respective food parameter effect score obtained from the study of Shivappa et al. [20]. Then, we calculated the overall DIP score for each participant by summing up all foods' DIP scores. Finally, a low DIP score (more negative) indicated a less inflammatory diet, and a higher DIP score (more positive) indicated a more inflammatory diet. This method has also been used in our previous studies [22].

### 2.1. Assessment of Anthropometric Measures

The self-report questionnaire was used to collect anthropometric data of participants including waist circumference (WC), weight, and height. The body mass index (BMI) was calculated using the BMI formula (BMI = weight (kilograms)/height^2^ (meters)). Based on BMI ≥ 25 kg/m^2^, participants were identified as overweight or obese. In addition, abdominal obesity was defined for women and men based on WC ≥ 80 cm and WC ≥ 94 cm, respectively.

The validity of self-reported waist circumferences, height, and weight was examined in a pilot study on 200 participants from the same population. A comparison was made between the self-reported values of anthropometric indices and the measured values in the validation study. The correlation coefficients between self-reported WC, height, and weight and the corresponding measured values were 0.60 (*P* < 0.001), 0.83 (*P* < 0.001), and 0.95 (*P* < 0.001), respectively. The correlation coefficient for computed BMI from self-reported values and the one from measured values was 0.70 (*P* < 0.001). These data indicated that the self-reported values of anthropometric measures provide a reasonable measure for these indices.

Assessment of other variables: information about gender, age, education (university graduate and below that), marital status (single, married, widowed, and divorced), smoking status (current smokers, former smokers, and nonsmokers), family size (>4/≤4 members), home ownership (nonowner/owner), and breakfast consumption (nonskippers/skippers) was gathered using a pretested self-administered questionnaire. Breakfast skippers were people who consumed breakfast less than 4 times per week. The General Physical Activity Questionnaire (GPPAQ) was used to assess participants' physical activity levels [23]. In this questionnaire, an individual's current physical activity is assessed using a simple, four-level physical activity index (PAI). Individuals were divided into four groups: inactive (without physical activity), moderately inactive (<1 hour per week), moderately active (1 to 3 hours per week), and active (>3 hours per week). In this study, participants were classified into two groups: physically active (≥1 h/week) or physically inactive (<1 h/week).

Statistical analysis: first we classified participants based on tertile cutoff points of DIP score. General characteristics of study participants across tertiles of DIP score were expressed as means ± SDs for continuous variables and percentages for categorical variables. The chi-square test for categorical variables and ANOVA for continuous variables were used to examine the differences across tertiles. Dietary intakes of study participants across tertiles of DIP score were compared by using analysis of covariance (ANCOVA). We used binary logistic regression to estimate ORs and 95% CIs for the presence of general and abdominal obesity across tertiles of DIP score in the crude and multivariable-adjusted models. In these analyses, total energy intake (continuous), sex (female/male), and age (continuous) were controlled for in the first model. Further adjustments were made for educational levels (university graduate and below that), physical activity (<1 h/week/≥1 h/week), marital status (single, married, widowed, and divorced), family size (≤4/>4 members), smoking (current smokers, former smokers, and nonsmokers), breakfast skipping (skippers/nonskippers), and home ownership (owner/nonowner) in the second model. P for trends was determined by considering tertiles of DIP score as ordinal variables in the logistic regression analysis. All statistical analyses were done using the Statistical Package for Social Sciences (version 20; SPSS Inc.). *P* < 0.05 was considered statistically significant.

## 3. Results

Mean age of study participants was 36.8 ± 8.08 years. The prevalence of general and abdominal obesity was 46.5% (in men: 41.2%, in women: 54.4%) and 52.9% (in men: 37.5%, in women: 64.4%), respectively. Compared with those in the lowest tertile, participants in the highest tertile of the DIP score had lower BMI and WC, were younger, more likely to be male, university graduated, homeowner, and less likely to be physically active. No significant differences were found in terms of other variables across tertiles of the DIP score (Table 1).

Dietary intakes of study participants across tertiles of the DIP score are shown in Table 2. Participants in the top tertile of the DIP score had higher intakes of energy, carbohydrate, saturated fat, trans fat, niacin, thiamin, and caffeine and lower intakes of fat, protein, fiber, cholesterol, MUFA, PUFA, vitamin B12, vitamin B6, folic acid, riboflavin, vitamin A, vitamin C, vitamin D, vitamin E, *β*-carotene, zinc, Mg, Fe, tea, onion, and pepper compared to those in the bottom tertile.

Crude and multivariable-adjusted odds ratios (ORs) and 95% confidence intervals (CIs) for general obesity across tertiles of the DIP score are shown in Table 3. In the fully adjusted model, participants in the top tertile of the DIP score had lower odds of general obesity compared with those in the bottom tertile (OR: 0.68; 95% CI: 0.58–0.79). When we performed the analysis stratified by gender, the DIP score was not associated with odds of general obesity in men (OR: 0.79; 95% CI: 0.62–1.01). However, a significant inverse association was found between adherence to a proinflammatory diet and odds of general obesity in women (OR: 0.58; 95% CI: 0.46–0.72).

Crude and multivariable-adjusted ORs and 95% CIs for abdominal obesity across tertiles of the DIP score are provided in Table 4. After taking potential confounders into account, we found no significant association between the DIP score and odds of abdominal obesity (OR: 0.91; 95% CI: 0.74–1.09). In addition, we found no significant relationship between adherence to a proinflammatory diet and odds of abdominal obesity in either gender (for men (OR: 1.11; 95% CI: 0.83–1.47) and for women (OR: 0.79; 95% CI: 0.62–1.01)).

The mean DIP score among subjects with normal weight, overweight, and obese indicated a significant difference (*P* < 0.001) such that obese people had the lowest DIP score (−0.28 ± 1.51) than that in normal-weight individuals (0.13 ± 1.48). Also, people with abdominal obesity had a lower mean DIP score (−0.09 ± 1.60) than people without abdominal obesity (0.10 ± 1.56); this difference was significant (*P* < 0.001).

## 4. Discussion

In this cross-sectional study, we observed a significant inverse association between adherence to a proinflammatory diet and odds of general obesity. In the gender-stratified analysis, we found this significant association in women only. We found no significant association between the DIP score and odds of abdominal obesity.

The prevalence of obesity is increasing in the world [24]. Obesity is associated with increased risk of some chronic conditions such as diabetes [25], metabolic syndrome [26], cardiovascular disease, and stroke [27]. Considering the high prevalence and economic burden of overweight and obesity, finding modifiable risk factors is of high priority.

Recently, huge attention has been paid to the role of inflammation in the etiology of obesity. In this study, we found a significant inverse association between adherence to a proinflammatory diet and odds of general obesity. In contrast, a cross-sectional study showed that a more proinflammatory diet was significantly associated with increased risk of general and abdominal obesity [7]. In addition, a prospective cohort study revealed that consumption of a proinflammatory diet was positively linked to annual weight gain and higher risk of developing overweight and obesity [28]. Findings of a meta-analysis showed that adherence to a proinflammatory diet was associated with increased BMI and obesity [29]. Also, a recent narrative review reported that an association between higher dietary inflammatory index and increased risk of obesity [30]. It must be kept in mind that the cross-sectional design of our study prohibits inferring a causal link between adherence to a proinflammatory diet and obesity. Inverse causality must also be considered such that obese people might follow a healthier diet in an attempt to improve their weight status. In addition, in the gender-stratified analysis, the inverse relationship was found only in women which further confirms our hypothesis about inverse causality because women pay much more attention to their appearance than men.

We found no significant association between adherence to a proinflammatory diet and odds of abdominal obesity. Similar to our study, a meta-analysis suggested that a high DIP score was not associated with an increased WC [31]. Contrary to our findings, a cohort study showed that participants in the highest quartile of the DIP score had a higher risk of abdominal obesity [5]. In addition, a cross-sectional study reported that mean waist-to-hip ratio was greater among those in the highest category of DIP score than those in the lowest category [32]. Lack of finding an association in the current study might be explained by the under-reporting of dietary intakes, which is common in epidemiologic studies and highly prevalent among obese people. In addition, the cross-sectional design of the study, as mentioned above, might provide some further explanations.

This study has several strengths. Having a large sample size and considering a wide range of potential confounders in statistical analysis are among the strengths of this study. Our study has some limitations that must be considered when interpreting our findings. Due to the cross-sectional design of the present study, causality cannot be inferred. Therefore, prospective studies are needed to confirm our findings. In addition, participants with obesity may have reduced their dietary intakes to lose weight. Although we controlled the analysis for several confounders, residual confounding cannot be easily ignored. Additional controlling for other confounding variables such as menopausal status, hormone therapy, and psychological factors might be needed to reach an independent association between DIP score with general and abdominal obesity. In addition, despite the use of a validated FFQ for dietary assessment, some degree of measurement errors and misclassification may be occurred. Furthermore, it must be kept in mind that anthropometric measurements were collected using a self-reported questionnaire. However, the validity of self-reported weight, height, and WC was examined in a pilot study.

In conclusion, there was a significant inverse association between adherence to a proinflammatory diet and odds of general obesity. This was also seen among women. In addition, we observed no significant association between the DIP score and odds of abdominal obesity.

## Figures and Tables

**Table 1:** General characteristics of study participants across tertiles of DIP score^a^.

Variables	Tertiles of DIP score			*P* value^b^
	*T*_1_	*T*_2_	*T*_3_	
Age, y	38.01 ± 8.2	36.97 ± 8.01	35.69 ± 7.84	<0.001
Female, %	63.4	62.1	58.4	0.01
Married, %	83.4	83.1	82.3	0.77
University graduated, %	58.4	60.2	65.8	<0.001
Family size (>4 people), %	10.2	9.9	9.7	0.84
Current smoker, %	4.2	3	4	0.05
Physically active (≥1 h/week), %	39.1	31	29.9	<0.001
Breakfast skipping (≥4 times/week), %	78	76.9	75.3	0.14
Home ownership (nonowner), %	27.3	30.8	36.5	<0.001
BMI, kg/m^2^	25.46 ± 3.88	24.92 ± 3.83	24.48 ± 3.82	<0.001
Waist circumference, cm	84.59 ± 15.7	83.45 ± 16.05	83.25 ± 16.26	0.02
Overweight or obese^c^, %	51.8	46.4	41.4	<0.001
Abdominal obesity^d^, %	57.3	53	51.6	0.002

^a^Data are mean ± standard deviation (SD); ^b^obtained from ANOVA or chi-square test, where appropriate; ^c^defined as BMI  ≥ 25 kg/m^2^; ^d^defined as waist circumference  ≥ 80 cm for women and  ≥94 cm for men.

**Table 2:** Dietary intakes of study participants across tertiles of DIP score^a^.

Variables	Tertiles of DIP score			*P* value^b^
	*T*_1_	*T*_2_	*T*_3_	
Energy (kcal/d)	2296 ± 17.31	2233.77 ± 17.31	2585.22 ± 17.32	<0.001
Carbohydrate (g/d)	289.12 ± 1.02	284.77 ± 1.03	296.09 ± 1.03	<0.001
Fat (g/d)	99.35 ± 0.38	101.05 ± 0.38	97.20 ± 0.38	<0.001
Protein (g/d)	92.44 ± 0.30	89.59 ± 0.30	83.25 ± 0.30	<0.001
Fiber (g/d)	26.46 ± 0.10	22.61 ± 0.10	18.48 ± 0.10	<0.001
Cholesterol (mg/d)	271.67 ± 2.00	265.28 ± 2.01	232.33 ± 2.02	<0.001
MUFA (g/d)	39.11 ± 0.17	39.69 ± 0.17	37.98 ± 0.18	<0.001
PUFA (g/d)	29.58 ± 0.13	29.72 ± 0.14	27.69 ± 0.14	<0.001
Saturated fat (g/d)	22.61 ± 0.12	23.63 ± 0.12	23.90 ± 0.12	<0.001
Trans fat (g/d)	0.20 ± 0.003	0.22 ± 0.003	0.23 ± 0.003	<0.001
Vitamin B12 (*μ*g/d)	3.11 ± 0.02	3.09 ± 0.02	2.77 ± 0.02	<0.001
Vitamin B6 (mg/d)	2.27 ± 0.007	2.02 ± 0.007	1.69 ± 0.007	<0.001
Folic acid (*μ*g/d)	589.80 ± 2.51	553.53 ± 2.52	546 ± 2.54	<0.001
Niacin (mg/d)	24.70 ± 0.08	24.65 ± 0.08	25.01 ± 0.08	0.008
Riboflavin (mg/d)	1.95 ± 0.009	1.85 ± 0.009	1.74 ± 0.009	<0.001
Thiamin (mg/d)	1.74 ± 0.01	1.76 ± 0.01	1.88 ± 0.01	<0.001
Vitamin A (RE)	670.53 ± 3.77	511.39 ± 3.78	391.49 ± 3.80	<0.001
Vitamin C (mg/d)	137.99 ± 0.95	98.99 ± 0.95	70.36 ± 0.95	<0.001
Vitamin D (*μ*g/d)	0.99 ± 0.01	1.00 ± 0.01	0.91 ± 0.01	<0.001
Vitamin E (mg/d)	23.22 ± 0.11	22.03 ± 0.11	19.36 ± 0.12	<0.001
*β*-Carotene (*μ*g/d)	5216.96 ± 33.61	3384.89 ± 33.71	2237.65 ± 33.95	<0.001
Caffeine (g/d)	101.92 ± 1.93	92.08 ± 1.93	105.59 ± 1.95	<0.001
Pepper (g/d)	5.96 ± 0.08	5.31 ± 0.08	3.85 ± 0.08	<0.001
Onion (g/d)	55.27 ± 0.57	41.52 ± 0.57	28.28 ± 0.57	<0.001
Tea (g/d)	446.86 ± 6.13	359.47 ± 6.15	299.52 ± 6.19	<0.001
Zn (mg/d)	11.74 ± 0.03	11.25 ± 0.03	10.25 ± 0.03	<0.001
Se (*μ*g/d)	105.98 ± 0.50	106.78 ± 0.51	105.16 ± 0.51	0.08
Mg (mg/d)	366.88 ± 0.96	329.16 ± 0.96	286.78 ± 0.97	<0.001
Fe (mg/d)	17.57 ± 0.06	17.16 ± 0.06	17.22 ± 0.06	<0.001

^a^Data are mean ± standard error (SE); ^b^all values were adjusted for age, sex, and energy, except for dietary energy intake, which was only adjusted for age and sex using ANCOVA.

**Table 3:** Crude and multivariable-adjusted odds ratio (95% CI) for general obesity across tertiles of DIP score^a^.

Variables	Tertiles of DIP score			*P*-trend
	*T*_1_	*T*_2_	*T*_3_	
Whole population				
Subjects, *n*	2241	2242	2241	
Crude	1.00	0.83 (0.74–0.93)	0.66 (0.58–0.74)	<0.001
Model I^b^	1.00	0.82 (0.73–0.92)	0.65 (0.58–0.73)	<0.001
Model II^c^	1.00	0.81 (0.69–0.95)	0.68 (0.58–0.79)	<0.001
Men				
Crude	1.00	0.87 (0.72–1.05)	0.78 (0.64–0.94)	0.01
Model I^d^	1.00	0.87 (0.72–1.05)	0.79 (0.65–0.95)	0.01
Model II^c^	1.00	0.83 (0.65–1.05)	0.79 (0.62–1.01)	0.06
Women				
Crude	1.00	0.78 (0.67–0.91)	0.57 (0.48–0.66)	<0.001
Model I^d^	1.00	0.78 (0.67–0.91)	0.58 (0.49–0.68)	<0.001
Model II^c^	1.00	0.80 (0.65–0.98)	0.58 (0.46–0.72)	<0.001

^a^Data are OR (95% CI). ^b^Model I: adjusted for age, sex, and energy intake. ^c^Model II: additionally, adjusted for marital status, education, family size, smoking status, physical activity, breakfast skipping, and home ownership. ^d^Model I: adjusted for age and energy intake.

**Table 4:** Crude and multivariable-adjusted odds ratio (95% CI) for abdominal obesity across tertiles of DIP score^a^.

Variables	Tertiles of DIP score			*P*-trend
	*T*_1_	*T*_2_	*T*_3_	
Whole population				
Subjects, *n*	1739	1740	1740	
Crude	1.00	0.83 (0.73–0.95)	0.79 (0.69–0.90)	0.001
Model I^b^	1.00	0.84 (0.73–0.97)	0.86 (0.74–0.99)	0.03
Model II^c^	1.00	0.88 (0.73–1.06)	0.91 (0.75–1.09)	0.31
Men				
Crude	1.00	0.91 (0.72–1.14)	1.05 (0.84–1.31)	0.60
Model I^d^	1.00	0.90 (0.72–1.14)	1.08 (0.87–1.36)	0.43
Model II^c^	1.00	0.90 (0.67–1.20)	1.11 (0.83–1.47)	0.44
Women				
Crude	1.00	0.81 (0.67–0.97)	0.71 (0.59–0.85)	<0.001
Model I^d^	1.00	0.80 (0.67–0.96)	0.74 (0.61–0.89)	0.001
Model II^c^	1.00	0.88 (0.69–1.12)	0.79 (0.62–1.01)	0.07

^a^Data are OR (95% CI). ^b^Model I: adjusted for age, sex, and energy intake. ^c^Model II: additionally, adjusted for marital status, education, family size, smoking status, physical activity, breakfast skipping, and home ownership. ^d^Model I: adjusted for age and energy intake.

## Data Availability

The dataset used and analyzed during the current study is available from the corresponding author on a reasonable request.
